# Association Between Anemia and In-Hospital Outcomes in Patients With ST-Segment Elevation Myocardial Infarction at King Abdullah Medical City

**DOI:** 10.7759/cureus.78859

**Published:** 2025-02-11

**Authors:** Fatma Aboul-Enein, Sara H Alharbi, Alaaddin Abdullah, Ahmad Aleissi, Fahad Almutairi, Fatmah M Alshariff, Rana Alsulami, Yosra Turkistani

**Affiliations:** 1 Internal Medicine and General Cardiology, King Abdullah Medical City, Makkah, SAU; 2 Cardiology, Alexandria University, Alexandria, EGY; 3 Internal Medicine, General Cardiology, and Advanced Cardiac Imaging, King Abdullah Medical City, Makkah, SAU; 4 Medical School, Umm Al-Qura University, Makkah, SAU; 5 Medical School, Kuwait University, Jabriya, KWT; 6 Medical School, Ibn Sina National College for Medical Studies, Jeddah, SAU

**Keywords:** anemia, length of stay, myocardial infarction, prognosis, stemi

## Abstract

Objective: This study aimed to assess the association between hemoglobin levels and in-hospital outcomes in patients with ST-segment elevation myocardial infarction (STEMI) at King Abdullah Medical City (KAMC).

Methods: This was a retrospective cohort study of 1,027 consecutive STEMI patients, from July 2022 to March 2023. The cohort was divided into two groups according to the presence of anemia on admission. Anemia was defined as hemoglobin levels of 12 g/dL in women and 13 g/dL in men: group 1 with anemia (255 (24.8%)) and group 2 without anemia (772 (75.2%)). Multiple logistic regression was used to examine the factors affecting in-hospital complications.

Results: Two hundred fifty-five (24.8%) patients were anemic at admission. Anemia was higher in women than men (49.7% vs. 20.5%; p < 0.001). The anemic group was older (59.5 ± 12.97 vs. 53.3 ± 11.76 years; p < 0.001) and had a higher prevalence of hypertension and diabetes mellitus (DM) (63.5% vs. 44.6% (p < 0.001) and 65.9% vs. 55.7% (p = 0.005), respectively). The anemic group had longer hospital stays (6.5 ± 7.4 vs. 4.77 ± 4.9 days; p < 0.001) and a higher in-hospital death rate (5.9% vs. 2.5%; p = 0.014). Using multivariate analysis, anemia was not a significant independent predictor for in-hospital complications.

Conclusion: Anemia is a common comorbidity in STEMI patients. It is associated with a 2.5-fold increase in in-hospital mortality and a significantly longer length of hospitalization.

## Introduction

ST-segment elevation myocardial infarction (STEMI) is a serious condition described as an occlusion in one or more of the coronary arteries leading to myocardial infarction, and it is considered a major cause of mortality and morbidity worldwide. On a global level, between 2007 and 2017, deaths from ischemic heart disease increased from 7.30 million (95% uncertainty interval (UI): 7.22-7.46) to 8.93 million (95% UI: 8.79-9.14). Anemia is common in Saudi Arabia; in a recent study of hospital attendees in Makkah, anemia has a prevalence of 38.7% and has multiple etiologies. Factors such as ethnic origin, smoking, dietary habits, and altitude of residence all influence red cell characteristics [1].

Incidental finding of anemia in patients with STEMI is a frequent condition and is considered a potent predictor of in-hospital outcomes. However, the mechanisms by which anemia affects the prognosis of STEMI patients remain unknown. Several mechanisms have been suggested, including disturbances in oxygen supply and demand, increases in catecholamine, and impairment of vascular circulation. These factors increase the risk of in-hospital major adverse cardiac events (MACE), such as in-hospital death, heart failure, pulmonary edema, cardiac arrest, cardiogenic events, shock, and the need for intubation or ventilation [2]. Moreover, common risk stratification tools that are used to guide management, such as the Global Registry of Acute Coronary Events (GRACE), do not include anemia as a prognostic factor; therefore, the aim of our study was to investigate the effects of anemia on the outcomes of STEMI patients.

Anemia is frequently observed in STEMI patients; however, it affects in-hospital outcomes, mortality, and complications and remains inadequately addressed in current research. This gap underscores the need to explore the risk factors and in-hospital clinical outcomes related to anemia among STEMI patients in tertiary centers in Saudi Arabia.

## Materials and methods

We conducted an observational retrospective cohort study to investigate the adverse outcomes of anemia on patients with STEMI at King Abdullah Medical City (KAMC) in Makkah after obtaining approval from the institute's Institutional Review Board (KAMC IRB) (approval number: 22-963). We included all patients who were admitted to the cardiology department over the age of 18 with STEMI. Patients with incomplete medical records were excluded (e.g., those with missing admission hemoglobin data). The study period was from July 2022 to March 2023, and a total of 1,027 patients were included. The sample size was calculated with the assumption of alpha = 0.05 and beta = 0.20 and an expected proportion in unexposed subjects of 0.6. Based on these assumptions, the estimated total sample size to provide sufficient power was 463.

Anemia was defined in the study as hemoglobin levels of <12 g/dL in women and <13 g/dL in men. All data were collected from patients' files. Patients were divided into two groups: group 1 with anemia (255 (24.8%)) and group 2 without anemia (772 (75.2%)). 

A composite endpoint of in-hospital complication is the occurrence of any cardiac complication including cardiogenic shock, cardiac arrest, intubation/ventilation, in-hospital death, further percutaneous coronary intervention (PCI) or coronary artery bypass grafting (CABG), left ventricular thrombus, cerebrovascular accident, pulmonary edema, low left ventricular ejection fraction (LVEF ≤40%), and major bleeding (defined as a drop in hemoglobin greater than 3 g/dL) [3].

Statistical analysis

For the data analysis, IBM SPSS Statistics for Windows, V. 26.0 (IBM Corp., Armonk, NY, USA), was used. Descriptive statistics were obtained to summarize the data and synthesize and report the variables. The numerical data were presented as means ± SDs or as medians and ranges according to the type of distribution of each variable. For the categorical variables, percentages and frequencies were assessed. Comparisons between groups were made by conducting a t-test or the Mann-Whitney U test. A chi-squared test was used to determine the association between the categorical variables. A p-value of less than 0.05 was considered significant, and the confidence interval (CI) was set at 95%.

## Results

A total of 1,027 consecutive patients who met the inclusion criteria were included in the study. Anemia was present in 255 (24.8%) of the total patients. The baseline clinical characteristics are summarized in Table 1.

**Table 1:** Baseline demographics and clinical characteristics of STEMI patients with anemia and without anemia. A p-value of less than 0.05 is considered significant. BMI: body mass index; CKD: chronic kidney disease; DM: diabetes mellitus; HGB: hemoglobin; HTN: hypertension; IHD: ischemic heart disease; LVEF: left ventricular ejection fraction; PCI-CABG: percutaneous coronary intervention-coronary artery bypass grafting; SD: standard deviation; STEMI: ST-segment elevation myocardial infarction

Variable		With anemia (N = 255 (24.8%))	Without anemia (N = 772 (75.2%))	P-value
Demographic				
Age (mean ± SD)		59.53 ± 12.98	53.25 ± 11.76	<0.001
Gender	Male	179 (70.2%)	695 (90.0%)	<0.001
	Female	76 (29.8%)	77 (10.0%)	
Pilgrim		24 (9.4%)	48 (6.2%)	0.112
BMI (mean ± SD)		27.73 ± 4.95	28.27 ± 5.07	0.137
HGB at admission (mean ± SD)		11.15 ± 1.50	14.83 ± 1.34	<0.001
Comorbidities				
DM		168 (65.9%)	430 (55.7%)	0.005
HTN		162 (63.5%)	344 (44.6%)	<0.001
Smoking	Current smoker	56 (22.0%)	249 (32.3%)	<0.001
	Ex-smoker	34 (13.3%)	157 (20.3%)	
	Non-smoker	165 (64.7%)	366 (47.4%)	
CKD		13 (5.1%)	24 (3.1%)	0.199
Dyslipidemia		15 (5.9%)	44 (5.7%)	1.000
Known IHD		11 (4.3%)	44 (5.7%)	0.489
Previous PCI-CABG		7 (2.7%)	34 (4.4%)	0.323
LVEF (mean ± SD)		39.29 ± 11.73	40.02 ± 10.40	0.380
Types of STEMI	Anterior	137 (53.7%)	479 (62.0%)	0.009
	Inferior	96 (37.6%)	259 (33.5%)	
	Other	22 (8.7%)	34 (4.5%)	
Lytic therapy		9 (3.5%)	36 (4.7%)	0.555

The anemic cohort was older, more frequently females; however, there was no significant difference between pilgrims (visitors to religious sites) and residents. Furthermore, the anemic group had a higher prevalence of hypertension (162 (63.5%) versus 344 (44.6%); p < 0.001) and diabetes mellitus (DM) (168 (65.9%) versus 430 (55.7%); p = 0.005). Regarding smoking status, most of the anemic patients were non-smokers (165 (64.7%) versus 366 (47.4%); p < 0.001). It was also found that non-anemic patients had anterior STEMI more often than the anemic group (479 (62%) versus 137 (53.7%); p = 0.009). In the distribution of hemoglobin values, the mean hemoglobin of the total population was 13.9 ± 2.1 g/dL, while the mean hemoglobin of the anemic patients was 11.14 ± 1.5 g/dL, which was lower than that of the non-anemic patients at 14.82 ± 1.33 g/dL (p < 0.001). The baseline demographics and clinical characteristics of STEMI patients with and without anemia are shown in Table 1.

Outcome characteristics

There was no significant relationship between anemia and the combined outcome, which included cardiogenic shock, cardiac arrest, intubation/ventilation, further PCI or CABG, left ventricular thrombus, cerebrovascular accident, pulmonary edema, low ejection fraction (LVEF ≤40%), major bleeding, and death, with a composite complication rate of 68.2% (174) in the anemic group compared to 63.5% (490) in the non-anemic group (p = 0.192). The in-hospital mortality rate was significantly greater in the anemic group, reaching 5.9% (15) versus 2.5% (19) (p = 0.014). Furthermore, the anemic group experienced longer hospital stays, with a mean duration of 6.48 ± 7.4 days compared to 4.77 ± 4.9 days in the non-anemic group (p < 0.001). In-hospital outcomes in STEMI patients with and without anemia are shown in Table 2.

**Table 2:** In-hospital outcomes in STEMI patients with anemia and without anemia. A p-value of less than 0.05 is considered significant. CVA: cerebrovascular accident; EF: ejection fraction; LV: left ventricular; LVEF: left ventricular ejection fraction; PCI-CABG: percutaneous coronary intervention-coronary artery bypass grafting; SD: standard deviation; STEMI: ST-segment elevation myocardial infarction

Variable	With anemia (N = 255 (24.8%))	Without anemia (N = 772 (75.2%))	P-value
CVA	9 (3.5%)	14 (1.8%)	0.173
Pulmonary edema	6 (2.4%)	8 (1.0%)	0.125
Cardiogenic shock	11 (4.3%)	20 (2.6%)	0.237
Intubation/ventilation	5 (2.0%)	9 (1.2%)	0.354
Cardiac arrest	5 (2.0%)	10 (1.3%)	0.546
Major bleeding	10 (3.9%)	59 (7.6%)	0.056
In-hospital death	15 (5.9%)	19 (2.5%)	0.014
LV thrombus	6 (2.4%)	11 (1.4%)	0.393
Further PCI or CABG	35 (13.7%)	78 (10.1%)	0.137
Low EF (LVEF ≤ 40%)	143 (56.1%)	424 (54.9%)	0.803
Composite	174 (68.2%)	490 (63.5%)	0.192
Length of stay (median)	4	3	<0.001

Multivariate logistical regression model

Table 3 outlines that anemia is a predictor of in-hospital mortality; however, after adjusting for other known predictors, only age and LVEF remain the independent predictors. 

**Table 3:** Univariate and multivariate binary regression analysis for predicting in-hospital mortality. DM: diabetes mellitus; CAD: coronary artery disease; LVEF: left ventricular ejection fraction; B: coefficient; S.E.: standard error; Sig.; p-value significance; Exp(B): odds ratio

	B	S.E.	Wald	Sig.	Exp(B)
Univariate regression analysis					
Anemia	0.976	0.347	7.892	0.005	2.65
Multivariate regression analysis					
Gender	-0.290	0.497	0.342	0.559	0.75
Age	0.028	0.014	3.902	0.048	1.03
DM	-0.287	0.387	0.551	0.458	0.75
Multivessel CAD	0.543	0.672	0.652	0.419	1.72
LVEF	-0.109	0.017	42.321	0.000	0.89
Anemia	0.570	0.398	2.052	0.152	1.77

## Discussion

Introduction

To our knowledge, this is the first study in Saudi Arabia to investigate anemia in STEMI patients. Our data demonstrated that anemia upon admission was common, occurring in one in four of all patients and one in three pilgrims, and was associated with higher comorbidities. Most importantly, we have demonstrated that anemia in STEMI leads to an over twofold increase in mortality. Furthermore, anemia was found to be associated with significantly longer hospital stays.

Comparison with previous studies

Although the patient cohort of the present study was over a decade younger than that of previously published studies, our data concur in demonstrating that anemia is a common comorbidity in acute coronary syndrome (ACS) patients, with a wide prevalence of 25% [4-6]. Similar to prior studies, our STEMI patients with anemia had higher comorbidities, including age, female gender, and DM, which suggests that anemia could be a marker of disease severity or complexity [6].

In-hospital complications and mortality

In the present study, patients with anemia tended to have a higher mortality and composite in-hospital complication rate (68.3% versus 63.5%), highlighting that baseline anemia may play a role in placing STEMI patients at a higher risk of complications. Moreover, anemic patients had a 2.5-fold increase in in-hospital mortality (5.9% versus 2.5%). This was also observed in prior studies conducted in the Middle East [7]. However, other studies have not revealed such statistically significant relationships [8,9]. This complex interrelation is multifactorial, as anemia may worsen ischemia through two main mechanisms: decreasing the oxygen supply and content to the jeopardized myocardium and increasing myocardial oxygen demand due to higher cardiac output [5]. Interestingly, literature has demonstrated that blood transfusion in anemic patients with or without hemorrhagic complications has also been shown to be a predictor of worse outcomes in ACS [10,11].

Admission hemoglobin levels and mortality

Although several studies have found a significant correlation between admission hemoglobin levels and in-hospital mortality in STEMI patients receiving primary PCI [12,13], other recent studies have shown no association after controlling for all the possible confounders [6,14]. Furthermore, Zeren et al. recently demonstrated that hemoglobin levels 24 hours post-PCI were a better predictor of in-hospital mortality than admission hemoglobin levels [14].

Anemia and bleeding risk paradox

In our study, an intriguing paradox emerged: despite anemia being linked to a 2.5-fold increase in in-hospital mortality, the anemic group surprisingly had fewer bleeding rates, with a tendency to be significant (p = 0.56). This paradox underscores the complex relationship between anemia, bleeding risk, and mortality in STEMI cases, highlighting the need for tailored management strategies. It may be explained in part that severe bleeding is defined per the Bleeding Academic Research Consortium guidelines, which do not take into consideration baseline hemoglobin level. Notably, our cohort is over a decade younger than other studies which may explain the paradox.

Clinical implications

Our study demonstrates the importance of assessing admission hemoglobin levels in STEMI patients, and particular attention should be paid to patients with low levels. The use of a glycoprotein IIb/IIIa inhibitor according to baseline renal function, the optimal dosing of unfractionated heparin or low-molecular-weight heparin, as well as the application of gastro-protective drugs are practical options for such patients. In addition, the radial vascular route should be the primary choice instead of the femoral vascular route to decrease blood loss following primary PCI [14].

Strengths of the study

In our study, we investigated the association between anemia and in-hospital outcomes in STEMI patients. We included a large consecutive group of patients, which strengthens the generalizability of our findings and reduces bias. Furthermore, by analyzing the patients' characteristics at the beginning of the study, we were able to interpret the results more accurately. One of the strengths of our study is the use of a powerful statistical method to identify independent risk factors for complications. This method helped us find that factors like age, gender, DM, and high blood pressure are important contributors to poor outcomes.

Limitations of the study

Our study has some limitations. Firstly, it is a retrospective study, so only the association between anemia and complications can be established. Secondly, it is a single-center study. Importantly, anemia was not a significant predictor of complications, suggesting other factors might be more influential. Finally, the lack of information regarding the severity of anemia was limiting our understanding of how it impacts outcomes. A multicenter prospective study would help identify these gaps.

## Conclusions

Our study highlights the importance of considering anemia as a risk factor in STEMI patients. Anemia is a common comorbidity in STEMI patients and is associated with a 2.5-fold increase in in-hospital mortality and a significantly longer length of hospitalization. Consequently, healthcare providers should remain vigilant about the potential impact of anemia when treating STEMI patients. Further research and examination of these relationships are needed to enhance our understanding of the complex factors influencing patient outcomes in STEMI cases.
